# Supplementary material for: Effect of Digital-Based Self-Learned Educational Intervention about COVID-19 Using Protection Motivation Theory on Non-Health Students’ Knowledge and Self-Protective Behaviors at Saudi Electronic University
Source: Int J Environ Res Public Health. 2022 Nov 8;19(22):14626. doi: 10.3390/ijerph192214626 (PMC9690823; doi:10.3390/ijerph192214626)
Supplement: Supplementary file 1 [file ijerph-19-14626-s001.zip › ijerph-2001976-supplementary.pdf]

# Non-Health Students' Knowledge and Self-Protective Behaviors Against COVID-19 Questionnaire

## Part 1: DEMOGRAPHIC DATA

1. Age (in years) ………………………………………………………………………
2. Gender: 1. Male 2. Female
3. Marital status: 1. Married 2. Divorced 3. Single
4. Academic level: ……………………………………………
5. Occupation: 1. Governmental 2. Private 3. Free business 4. Not working
6. City of residence 1. Riyadh 2. Dammam 3. Jeddah
7. Have you worked in a healthcare facility? 1. Yes 2. No
8. Do you know anyone who has been diagnosed in your circle with corona in this year? 1. Yes 2. No

## Part 2: KNOWLEDGE ABOUT CORONA INFECTION

9. Is Corona a viral infection? Yes ☐ No ☐ Don't know ☐
10. Can the same person catch COVID-19 infection more than one time? Yes ☐ No ☐ Don't know ☐
11. How does Corona infection spread?

| | Yes | No | Don't know |
|---|---|---|---|
| a. Shaking hands with infected person | ☐ | ☐ | ☐ |
| b. Droplet of sneeze and cough | ☐ | ☐ | ☐ |
| c. Imported products from China | ☐ | ☐ | ☐ |
| d. Contaminated surfaces or objects | ☐ | ☐ | ☐ |

12. Fever, cough, cold and shortness of breath are symptoms of corona infection. Yes ☐ No ☐ Don't know ☐
13. Symptoms appear 2-14 days after the infection Yes ☐ No ☐ Don't know ☐

14. Groups who are at increased risk of corona infection

| | Yes | No | Don't know |
|---|---|---|---|
| a. Children less than 5 years | ☐ | ☐ | ☐ |
| b. People with chronic disease | ☐ | ☐ | ☐ |
| c. Elderly patients | ☐ | ☐ | ☐ |
| d. People with poor immunity | ☐ | ☐ | ☐ |

15. Which of the following can help in preventing corona infection?

| | Yes | No | Don't know |
|---|---|---|---|
| a. Washing hands with soap | ☐ | ☐ | ☐ |
| b. Using hand sanitizer | ☐ | ☐ | ☐ |
| c. Wearing a face mask | ☐ | ☐ | ☐ |
| d. Covering nose while sneezing | ☐ | ☐ | ☐ |
| e. Staying at home | ☐ | ☐ | ☐ |

16. When should one seek emergency medical attention?

| | Yes | No | Don't know |
|---|---|---|---|
| a. Difficulty in breathing | ☐ | ☐ | ☐ |
| b. Persistent pain or pressure in chest | ☐ | ☐ | ☐ |
| c. Bluish lip or face | ☐ | ☐ | ☐ |
| d. Confusion | ☐ | ☐ | ☐ |
| e. Fatigue | ☐ | ☐ | ☐ |

17. what are the benefits of COVID-19 vaccine?

| | Yes | No | Don't know |
|---|---|---|---|
| a. Reduce the risk for infection | ☐ | ☐ | ☐ |
| b. Reduce the severity of symptoms | ☐ | ☐ | ☐ |
| c. Reduce the incidence of sever complication | ☐ | ☐ | ☐ |
| d. Reduce mortality rate | ☐ | ☐ | ☐ |

| | Yes | No | Don't know |
|---|---|---|---|
| 18. Can COVID-19 vaccine provide long life immunity? | ☐ | ☐ | ☐ |

19. What are your Sources of information about COVID-19 pre-intervention? (More than one source can be chosen)

1. Saudi Ministry of Health
2. Health Care Staff
3. International Health Websites
4. Official Saudi Health websites
5. Peer Reviewed Health Journals
6. Mass media
7. Social media
8. Friends and Relatives

## Part 3: Self-Protective Behaviors Scale

| ***Question*** | **To what extent you are carful to do the following?** | **Always** | **Occasionally** | **Never** |
|---|---|---|---|---|
| 20. | Wearing facemask | | | |
| 21. | Social distancing | | | |
| 22. | Hand washing or alcohol rub. | | | |
| 23. | Disinfect surfaces | | | |
| 24. | Respiratory etiquette | | | |
| 25. | Healthy lifestyle | | | |
| 26. | Hospital visiting for emergency signs | | | |
| 27. | Vaccination. | | | |

## Part 4: Protection Motivation Theory (PMT) Constructs Scale

| Ques | Statement | Strongly Agree | Agree | Neutral | Disagree | Strongly Disagree |
|---|---|---|---|---|---|---|
| **A.** | **Perceived vulnerability** | | | | | |
| 1. | I am more susceptible than others to catch COVID-19 infection. | | | | | |
| 2. | My health status makes me vulnerable to COVID-19 infection. | | | | | |
| 3. | I am susceptible to COVID-19 infection due to unhealthy lifestyle and negligence of protective measures inside and outside the home | | | | | |
| **B.** | **Perceived severity** | | | | | |
| 4. | COVID-19 infection may lead to severe pneumonia and respiratory failure | | | | | |
| 5. | COVID-19 infection may cause severe and permanent lung damage | | | | | |
| 6. | COVID-19 infection is highly contagious and may infect the whole family | | | | | |
| **C.** | **Fear** | | | | | |
| 7. | It terrifies me to think that I may be infected with COVID-19. | | | | | |
| 8. | The idea of being isolated in the hospital terrifies me | | | | | |
| 9. | I feel very awful if I notice any symptoms like those of COVID-19. | | | | | |
| **D.** | **Intrinsic reward** | | | | | |
| 10. | If I adhere to the preventive measures, it will reduce my anxiety about COVID-19 infection. | | | | | |
| 11. | Adhering to a healthy lifestyle helps me raise my immunity and protect me from COVID-19 infection. | | | | | |
| 12. | Social distancing greatly reduces my risk and stress related to COVID-19 infection. | | | | | |

| Ques | Statement | Strongly Agree | Agree | Neutral | Disagree | Strongly Disagree |
|---|---|---|---|---|---|---|
| **E.** | **Extrinsic reward** | | | | | |
| 13. | My family encourages me to adhere to preventive measures and social distancing | | | | | |
| 14. | My family encourages me to adhere to a healthy lifestyle to prevent COVID-19 infection. | | | | | |
| 15. | The health team provides me with the necessary instructions to prevent COVID-19 infection. | | | | | |
| **F.** | **Response efficacy** | | | | | |
| 16. | Adhering to preventive measures and social distancing can save my life | | | | | |
| 17. | COVID-19 vaccine may protect me from infection. | | | | | |
| 18. | If I stick to the healthy lifestyles, I will be safe from COVID-19 infection. | | | | | |
| **G.** | **Self-efficacy** | | | | | |
| 19. | I am confident that I will be able to deal efficiently with COVID-19. | | | | | |
| 20. | My ability to adapt helps me stay calm in difficult situations while fighting the virus | | | | | |
| 21. | If I am in trouble, I can usually think of appropriate solutions and alternatives to stay safe from infection | | | | | |
| **H.** | **Response cost** | | | | | |
| 22. | Despite the importance of preventive measures, it makes me feel embarrassed* | | | | | |
| 23. | Preventive measures are costly* | | | | | |
| 24. | If I adhere to social distancing and preventive measures, others will bully me* | | | | | |

| Ques | Statement | Strongly Agree | Agree | Neutral | Disagree | Strongly Disagree |
|---|---|---|---|---|---|---|
| **I.** | **Behavior intention** | | | | | |
| 25. | I intend to apply social distancing as a preventive measure, no matter what it costs me. | | | | | |
| 26. | I intend to seek health care if I suffered from COVID-19 symptoms. | | | | | |
| 27. | I intended to change my lifestyle to a healthy one. | | | | | |
| 28. | I am intended to take COVID-19 vaccines regardless of its risk. | | | | | |
